# An improved approach for studying oscillation of second-order neutral delay differential equations

**DOI:** 10.1186/s13660-018-1767-y

**Published:** 2018-07-27

**Authors:** Said R. Grace, Jozef Džurina, Irena Jadlovská, Tongxing Li

**Affiliations:** 1 Department of Engineering Mathematics, Faculty of Engineering, Cairo University, Giza, Egypt; 2 Department of Mathematics and Theoretical Informatics, Faculty of Electrical Engineering and Informatics, Technical University of Košice, Košice, Slovakia; 3 School of Information Science and Engineering, Linyi University, Linyi, P.R. China; 4 School of Control Science and Engineering, Shandong University, Jinan, P.R. China

**Keywords:** 34C10, 34K11, Neutral differential equation, Delayed argument, Second-order, Riccati substitution, Oscillation

## Abstract

The paper is devoted to the study of oscillation of solutions to a class of second-order half-linear neutral differential equations with delayed arguments. New oscillation criteria are established, and they essentially improve the well-known results reported in the literature, including those for non-neutral differential equations. The adopted approach refines the classical Riccati transformation technique by taking into account such part of the overall impact of the delay that has been neglected in the earlier results. The effectiveness of the obtained criteria is illustrated via examples.

## Introduction

In this paper, we are concerned with the oscillation problem of a class of second-order half-linear neutral delay differential equations 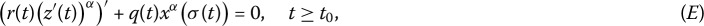 where $z(t) := x(t)+p(t)x(\tau(t))$. Throughout the paper, we always assume that

(H_1_): *α* is a quotient of odd positive integers;
(H_2_): $r\in \mathrm{C}^{1}([t_{0},\infty),(0,\infty))$, $p, q \in\mathrm{C}([t_{0},\infty),[0,\infty))$, $0\le p(t)< 1$, and $q(t)$ does not vanish identically on any half-line of the form $[t_{*},\infty )$, $t_{*}\ge t_{0}$;
(H_3_): $\tau, \sigma\in\mathrm{C}([t_{0},\infty),\mathbb {R})$ satisfy $\tau(t)\le t$, $\sigma(t )< t$, and $\lim_{t\to\infty}\tau(t) =\lim_{t\to\infty}\sigma(t) = \infty$.

We will consider the following case:

1 $$ \int_{t_{0}}^{\infty}r^{-1/\alpha}(s)\,\mathrm {d}s =\infty $$

in order to complement the recent work [1], where the oscillatory behavior of (*E*) under the assumption

$$\int_{t_{0}}^{\infty}r^{-1/\alpha}(s)\,\mathrm {d}s < \infty $$

has been investigated.

By a solution of (*E*) we mean a nontrivial real-valued function $x\in\mathrm{C}([t_{a},\infty),\mathbb {R})$ with $t_{a} := \min\{\tau(t_{b}),\sigma (t_{b})\}$ for some $t_{b}\ge t_{0}$, which has the property $r (z' )^{\alpha}\in\mathrm{C}^{1}([t_{0},\infty),\mathbb {R})$ and satisfies (*E*) on $[t_{0},\infty)$. We consider only those solutions of (*E*) which exist on some half-line $[t_{b},\infty)$ and satisfy the condition

$$\sup\bigl\{ \bigl\vert x(t) \bigr\vert :t_{c}\le t< \infty\bigr\} >0 \quad \text{for any } t_{c}\geq t_{b}. $$

As is customary, a solution *x* of (*E*) is said to be oscillatory if it has arbitrarily large zeros; otherwise, it is said to be nonoscillatory. The equation itself is called oscillatory if all its solutions oscillate.

The oscillation theory of differential equations with deviating arguments was initiated in a pioneering paper [2] of Fite, which appeared in the first quarter of the twentieth century. Since then, there has been much research activity concerning the oscillation of solutions of various classes of differential and functional differential equations. The interest in this subject has been reflected by extensive references in monographs [3–7]. We also refer the reader to the papers [8–10] and the references cited therein regarding similar discrete analogues of (*E*) and its particular cases and modifications.

A neutral delay differential equation is a differential equation in which the highest order derivative of the unknown function appears both with and without delay. During the last three decades, oscillation of neutral differential equations has become an important area of research; see, e.g., [11–27]. This is due to the fact that such equations arise from a variety of applications including population dynamics, automatic control, mixing liquids, and vibrating masses attached to an elastic bar; see Hale [28]. Especially, second-order neutral delay differential equations are of great interest in biology in explaining self-balancing of the human body and in robotics in constructing biped robots [29].

One of the traditional tools in the study of oscillation of equations which are special cases of (*E*) has been based on a reduction of order and the comparison with oscillation of first-order delay differential equations. In particular, Koplatadze in 1986 [30] and Wei in 1988 [31] proved that the second-order delay differential equation

2 $$ x''(t)+q(t)x\bigl(\sigma(t)\bigr) = 0 $$

is oscillatory if 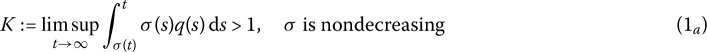 or 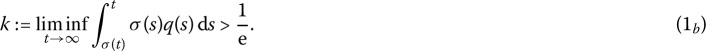 Conditions ($1_{a}$) and ($1_{b}$), which obviously hold for delay equations only, are analogous to the well-known oscillation criteria due to Ladas et al. [32] and Koplatadze and Chanturiya [33], respectively, 
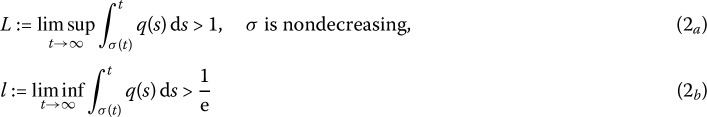
 for the first-order delay differential equation

3 $$ x'(t)+q(t)x\bigl(\sigma(t)\bigr) = 0. $$

There is an obvious gap between conditions ($1_{a}$)–($1_{b}$) (($2_{a}$)–($2_{b}$)) if $k< K$ ($l< L$). For first-order equations, filling this gap has been an interesting research problem in the last few decades; see, e.g., the excellent survey [34] and the references cited therein. In 2000, Koplatadze et al. [35] presented the following oscillation criteria for (2) which improve ($1_{a}$)–($1_{b}$), namely 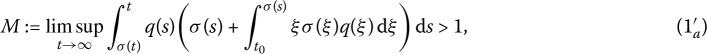 where *σ* is nondecreasing, or 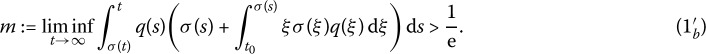 One may note that, despite similarities, there is a significant difference between ($1_{a}$)–($1_{b}$) (($1_{a}'$)–($1_{b}'$)) and ($2_{a}$)–($2_{b}$). According to [33], it is known that if $L< 1/\mathrm {e}$, then (3) has a nonoscillatory solution. Oscillation of equation (3) is caused by the presence of delay. However, equation (2) can be oscillatory even in the case where $\sigma(t)= t$.

Another widely used technique, applicable also in the above-mentioned case, involves the Riccati type transformation which has been used to reduce equation (*E*) to a first-order Riccati inequality. In 2006, Sun and Meng [36] improved the oscillation result of Džurina and Stavroulakis [37] by employing the Riccati transformation

$$w(t): = R^{\alpha}\bigl(\sigma(t)\bigr)\frac{r(t) (x'(t) )^{\alpha}}{x^{\alpha}(\sigma(t))}, \qquad R(t): = \int_{t_{1}}^{t}r^{-1/\alpha }(s)\,\mathrm {d}s, \quad t_{1}\ge t_{0} \text{ is large enough}, $$

which yields that the particular case of (*E*), equation 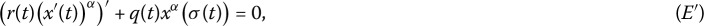 is oscillatory if (1) holds, $\sigma\in\mathrm{C}^{1}([t_{0},\infty ),\mathbb {R})$, $\sigma'(t)>0$, and

4 $$ \int^{\infty} \biggl(R^{\alpha}\bigl(\sigma(s)\bigr)q(s)- \biggl(\frac {\alpha}{\alpha+1} \biggr)^{\alpha+1}\frac{\sigma'(s)}{R(\sigma (s))r^{1/\alpha}(\sigma(s)) } \biggr)\,\mathrm {d}s= \infty. $$

Xu and Meng [23] generalized condition (4) to (*E*) and proved that if (1) is satisfied, $\sigma\in\mathrm{C}^{1}([t_{0},\infty),\mathbb {R})$, $\sigma'(t)>0$, and

5 $$ \int^{\infty} \biggl(R^{\alpha}\bigl(\sigma(s)\bigr)q(s) \bigl(1-p\bigl(\sigma (s)\bigr)\bigr)^{\alpha}- \biggl(\frac{\alpha}{\alpha+1} \biggr)^{\alpha+1}\frac {\sigma'(s)}{R(\sigma(s))r^{1/\alpha}(\sigma(s)) } \biggr)\,\mathrm {d}s= \infty, $$

then (*E*) is oscillatory. Later on, Erbe et al. [38] showed that ($E'$) is oscillatory assuming that (1) holds, $\alpha\ge1$, $r'\ge0$, $\int _{t_{0}}^{\infty}\sigma^{\alpha}(s)q(s)\,\mathrm {d}s =\infty$, and

6 $$ \limsup_{t\to\infty} \int_{t_{0}}^{t} \biggl( \psi(s) q(s) \biggl( \frac{\sigma(s)}{s} \biggr)^{\alpha}- \frac{ (\psi '_{+}(s) )^{\alpha+1}r(s)}{ (\alpha+1)^{\alpha+1}\psi^{\alpha}(s) } \biggr)\,\mathrm {d}s= \infty, $$

where $\psi\in\mathrm{C}^{1}([t_{0},\infty),(0,\infty))$ and $\psi '_{+}(t):=\max\{0, \psi'(t)\}$. The similar ideas as those above have been exploited and extended for (*E*) and its various generalizations in a number of papers; see, e.g., [11, 16, 17, 20–23, 26, 27, 39] and the references therein.

The objective of this paper is to establish new oscillation results for (*E*), which would improve the above-mentioned ones. The paper is organized as follows. First, motivated by [35], we generalize conditions ($1_{a}'$) and ($1_{b}'$) for linear equation (2) to be applicable to the half-linear neutral equation (*E*). Second, we refine classical Riccati transformation techniques to obtain new oscillation criteria, which, to the best of our knowledge, essentially improve a large number of related results reported in the literature, including those for second-order delay differential equations. The adopted approach lies in establishing sharper estimates relating a nonoscillatory solution with its derivatives in the case when conditions analogous to ($1_{a}'$)–($1_{b}'$) fail to apply. We illustrate the effectiveness of the obtained criteria via a series of examples and comparison with other known oscillation results.

In what follows, all occurring functional inequalities are assumed to hold eventually, that is, they are satisfied for all *t* large enough. As usual and without loss of generality, we can deal only with eventually positive solutions of (*E*).

## Preliminaries

For the sake of brevity and clarity, we let

$$\begin{aligned}& Q(t) : = \bigl(1-p\bigl(\sigma(t)\bigr)\bigr)^{\alpha}q(t), \\& \tilde{Q}(t) : = \int_{t}^{\infty}Q(s)\,\mathrm {d}s, \\& R(t) := \int_{t_{1}}^{t}r^{-1/\alpha}(s)\,\mathrm {d}s, \\& \tilde{R}(t) := R(t)+ \frac{1}{\alpha} \int_{t_{1}}^{t}R(s)R^{\alpha}\bigl(\sigma(s) \bigr)Q(s)\,\mathrm {d}s, \\& \hat{R}(t) :=\exp \biggl( -\alpha \int_{\sigma(t)}^{t}\frac{\mathrm{d} s}{\tilde{R}(s)r^{1/\alpha}(s)} \biggr) \end{aligned}$$

for $t\ge t_{1}$, where $t_{1}\ge t_{0}$ is large enough.

To prove our oscillation criteria, we need the following auxiliary results.

### Lemma 1

(see [12, Lemma 3])

*Let condition* (1) *hold and assume that* $x(t)$ *is a positive solution of* (*E*) *on* $[t_{0},\infty)$. *Then there exists a* $t_{1}\ge t_{0}$ *such that*, *for* $t\ge t_{1}$,

7 $$ z(t)>0,\qquad z'(t)>0, \qquad \bigl(r(t) \bigl(z'(t) \bigr)^{\alpha}\bigr)'\le0. $$

### Lemma 2

(see [27, Lemma 2.3])

*Let* $g(u) = Au - Bu^{(\alpha+1)/\alpha}$, *where* *A* *and* $B>0$ *are constants*, *α* *is a quotient of odd natural numbers*. *Then* *g* *attains its maximum value on* $\mathbb {R}$ *at* $u^{*} = (\alpha A/((\alpha +1)B) )^{\alpha}$ *and*

8 $$ \max_{u\in \mathbb {R}} g = g \bigl(u^{*} \bigr) = \frac{\alpha^{\alpha}}{(\alpha+1)^{\alpha+1}}\frac{A^{\alpha+1}}{B^{\alpha}}. $$

## Main results

Now, we state and prove our first oscillation result, which extends [35, Theorem 3] obtained for the linear delay differential equation (2) to the half-linear neutral delay differential equation (*E*).

### Theorem 3

*Let condition* (1) *be satisfied*. *If*

9 $$ \limsup_{t\to\infty} \int_{\sigma(t)}^{t}Q(s)\tilde {R}^{\alpha}\bigl( \sigma(s)\bigr)\,\mathrm {d}s>1, \quad \sigma \textit{ is nondecreasing} $$

*or*

10 $$ \liminf_{t\to\infty} \int_{\sigma(t)}^{t}Q(s)\tilde {R}^{\alpha}\bigl( \sigma(s)\bigr)\,\mathrm {d}s>\frac{1}{\mathrm {e}}, $$

*then* (*E*) *is oscillatory*.

### Proof

Assume that (*E*) has a nonoscillatory solution $x(t)$ on $[t_{0},\infty)$. Without loss of generality, we may assume that there exists a $t_{1}\ge t_{0}$ such that $x(t)>0$, $x(\tau(t))>0$, and $x(\sigma (t))>0$ for $t\ge t_{1}$. By the definition of $z(t)$, we obtain, for $t\ge t_{1}$,

$$x(t)\ge z(t) - p(t)x\bigl(\tau(t)\bigr)\ge z(t) - p(t)z\bigl(\tau(t)\bigr)\ge \bigl(1-p(t)\bigr)z(t), $$

which together with (*E*) implies that

11 $$ \bigl(r(t) \bigl(z'(t) \bigr)^{\alpha}\bigr)' \le- Q(t)z^{\alpha}\bigl(\sigma(t)\bigr). $$

On the other hand, it follows from the monotonicity of $r^{1/\alpha }(t)z'(t)$ that

12 $$ z(t) = z(t_{1})+ \int_{t_{1}}^{t}\frac{1}{r^{1/\alpha}(s)}r^{1/\alpha }(s)z'(s) \,\mathrm {d}s \ge R(t)r^{1/\alpha}(t)z'(t). $$

A simple computation shows that

13 $$ \bigl(z(t) - R(t)r^{1/\alpha}(t)z'(t) \bigr)' = - R(t) \bigl(r^{1/\alpha}(t)z'(t) \bigr)'. $$

Applying the chain rule, it is easy to see that

$$ R(t) \bigl(r(t) \bigl(z'(t) \bigr)^{\alpha} \bigr)' = \alpha R(t) \bigl(r^{1/\alpha}(t)z'(t) \bigr)^{\alpha-1} \bigl(r^{1/\alpha }(t)z'(t) \bigr)'. $$

By virtue of (11), the latter equality yields

14 $$ -R(t) \bigl(r^{1/\alpha}(t)z'(t) \bigr)' \ge\frac{1}{\alpha }R(t) \bigl(r^{1/\alpha}(t)z'(t) \bigr)^{1-\alpha}Q(t)z^{\alpha}\bigl(\sigma(t)\bigr). $$

Combining (13) and (14), we obtain

15 $$ \bigl( z(t) - R(t)r^{1/\alpha}(t)z'(t) \bigr)' \ge\frac{1}{\alpha}R(t) \bigl(r^{1/\alpha}(t)z'(t) \bigr)^{1-\alpha}Q(t)z^{\alpha}\bigl(\sigma(t)\bigr). $$

Integrating (15) from $t_{1}$ to *t*, we have

$$ z(t)\ge R(t)r^{1/\alpha}(t)z'(t)+\frac{1}{\alpha} \int _{t_{1}}^{t} \bigl(r^{1/\alpha}(s)z'(s) \bigr)^{1-\alpha }R(s)Q(s)z^{\alpha}\bigl(\sigma(s)\bigr)\,\mathrm {d}s. $$

Taking (12) and the monotonicity of $r^{1/\alpha}(t)z'(t)$ into account, we arrive at

16 $$\begin{aligned} z(t)&\ge R(t)r^{1/\alpha}(t)z'(t) \\ &\quad {} +\frac{1}{\alpha} \int _{t_{1}}^{t} \bigl(r^{1/\alpha}(s)z'(s) \bigr)^{1-\alpha}R(s)R^{\alpha}\bigl(\sigma(s)\bigr)Q(s)r\bigl( \sigma(s)\bigr) \bigl(z'\bigl(\sigma(s)\bigr) \bigr)^{\alpha} \,\mathrm {d}s \\ &\ge R(t)r^{1/\alpha}(t)z'(t) \\ &\quad {} +\frac{1}{\alpha} \int _{t_{1}}^{t} \bigl(r^{1/\alpha}(s)z'(s) \bigr)^{1-\alpha}R(s)R^{\alpha}\bigl(\sigma(s)\bigr)Q(s)r(s) \bigl(z'(s) \bigr)^{\alpha} \,\mathrm {d}s \\ &\ge r^{1/\alpha}(t)z'(t) \biggl(R(t)+ \frac{1}{\alpha} \int _{t_{1}}^{t}R(s)R^{\alpha}\bigl(\sigma(s) \bigr)Q(s)\,\mathrm {d}s \biggr). \end{aligned}$$

Thus, we conclude that

17 $$ z\bigl(\sigma(t)\bigr)\ge r^{1/\alpha}\bigl(\sigma(t) \bigr)z'\bigl(\sigma(t)\bigr) \tilde {R}\bigl(\sigma(t)\bigr). $$

Using (17) in (11), by virtue of (7), one can see that $y(t): = r(t) (z'(t) )^{\alpha}$ is a positive solution of the first-order delay differential inequality

18 $$ y'(t)+Q(t)\tilde{R}^{\alpha}\bigl(\sigma(t) \bigr)y\bigl(\sigma(t)\bigr)\le0. $$

In view of [40, Theorem 1], the associated delay differential equation

19 $$ y'(t)+Q(t)\tilde{R}^{\alpha}\bigl(\sigma(t) \bigr)y\bigl(\sigma(t)\bigr)= 0 $$

also has a positive solution. However, it is well known that condition (9) or condition (10) ensures oscillation of (19). This in turn means that (*E*) cannot have positive solutions. The proof is complete. □

Letting $p(t) = 0$ in (*E*), the following result is an immediate consequence.

### Corollary 1

*Let condition* (1) *hold*. *If*

20 $$ \limsup_{t\to\infty} \int_{\sigma(t)}^{t}q(s)\tilde {R}^{\alpha}\bigl( \sigma(s)\bigr)\,\mathrm {d}s>1, \quad \sigma \textit{ is nondecreasing} $$

*or*

21 $$ \liminf_{t\to\infty} \int_{\sigma(t)}^{t}q(s)\tilde {R}^{\alpha}\bigl( \sigma(s)\bigr)\,\mathrm {d}s>\frac{1}{\mathrm {e}}, $$

*then* ($E'$) *is oscillatory*.

### Remark 1

Note that for $\alpha= 1$, $r(t) = 1$, and $p(t)=0$, Theorem 3 reduces to [35, Theorem 3].

### Example 1

For $t\ge1$, consider the second-order neutral differential equation 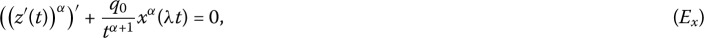 where $z(t): = x(t)+p_{0}x(\tau(t))$, *α* is a quotient of odd positive integers, $p_{0}\in[0,1)$, $\tau(t)\le t$, $q_{0}>0$, and $\lambda\in(0,1)$. By Theorem 3, ($E_{x}$) is oscillatory if 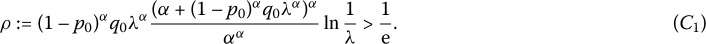 For a particular case of ($E_{x}$), equation

22 $$ \bigl( \bigl(x'(t) \bigr)^{1/3} \bigr)'+\frac{q_{0}}{t^{4/3}}x^{1/3}(0.9t)=0, $$

oscillation of all solutions is guaranteed by condition

23 $$ q_{0}>1.92916. $$

To the best of our knowledge, the known related criterion for (22) based on comparison with a first-order delay differential equation (see, e.g., [12, Theorem 2]) gives $q_{0}>3.61643$, which is a significantly weaker result.

On the other hand, for equation

24 $$ \bigl( \bigl(x'(t) \bigr)^{1/3} \bigr)'+\frac{1}{6} \biggl(\frac {5}{18} \biggr)^{1/6}t^{-4/3}x^{1/3}(0.9t)=0, $$

condition (23) fails to hold and $x(t) = t^{1/2}$ is a nonoscillatory solution of (24).

Obviously, if

25 $$ \int_{\sigma(t)}^{t}Q(s)\tilde{R}^{\alpha}\bigl( \sigma(s)\bigr)\,\mathrm {d}s \le \frac{1}{\mathrm {e}}, $$

then Theorem 3 cannot be applied to (*E*). However, if (25) holds and $y(t)$ is a positive solution of (18), then it is possible to obtain sharper lower bounds of the ratio ${y(\sigma(t))}/{y(t)}$. This will allow us to refine classical Riccati transformation techniques which are widely used in the study of oscillation of second-order differential equations. Zhang and Zhou [41] obtained such bounds for the first-order delay differential equation (19) by employing a sequence $\{ f_{n}(\rho)\}_{n = 0}^{\infty}$ defined as

26 $$ f_{0}(\rho) := 1, \qquad f_{n+1}(\rho) := \mathrm {e}^{\rho f_{n}(\rho)}, \quad n = 0,1,2,\ldots, $$

where *ρ* is a positive constant satisfying

27 $$ \int_{\sigma(t)}^{t}Q(s)\tilde{R}^{\alpha}\bigl( \sigma(s)\bigr)\,\mathrm {d}s \ge\rho , \quad t\ge t_{1}\ge t_{0}. $$

They showed that, for $\rho\in(0,1/\mathrm {e}]$, the sequence is increasing and bounded above and $\lim_{t\to\infty}f_{n}(\rho) = f(\rho)\in [1,\mathrm {e}]$, where $f(\rho)$ is a real root of the equation

28 $$ f(\rho) = \mathrm {e}^{\rho f(\rho)}. $$

Their result plays an essential role when proving the following lemma.

### Lemma 4

*Let condition* (1) *hold and assume that* *σ* *is strictly increasing*, *condition* (27) *holds for some* $\rho>0$, *and* (*E*) *has a positive solution* $x(t)$ *on* $[t_{0},\infty)$. *Then*, *for every* $n\ge0$, $y(t) := r(t) (z'(t) )^{\alpha}$ *satisfies*

29 $$ \frac{y(\sigma(t))}{y(t)}\ge f_{n}(\rho) $$

*for* *t* *large enough*, *where* $f_{n}(\rho)$ *is defined by* (26).

### Proof

Assume that (*E*) has a nonoscillatory solution $x(t)$ on $[t_{0},\infty)$. Without loss of generality, we can suppose that there exists a $t_{1}\ge t_{0}$ such that $x(t)>0$, $x(\tau(t))>0$, and $x(\sigma (t))>0$ for $t\ge t_{1}$. As in the proof of Theorem 3, we deduce that $y(t): = r(t) (z'(t) )^{\alpha}$ is a positive solution of the first-order delay differential inequality (18). Proceeding in a similar manner as in the proof of [41, Lemma 1], we see that estimate (29) holds. □

In what follows, we employ the Riccati substitution technique to obtain new oscillation criteria for (*E*), which are especially effective in the case when Theorem 3 fails to apply.

### Theorem 5

*Let condition* (1) *be satisfied and assume that* $\sigma\in \mathrm{C}^{1}([t_{0},\infty),\mathbb {R})$, $\sigma'(t)>0$, *and condition* (27) *holds for some* $\rho>0$. *If there exists a function* $\varphi\in \mathrm{C}^{1}([t_{0},\infty),(0,\infty))$ *such that*, *for some sufficiently large* $T\ge t_{1}$ *and for some* $n\ge0$,

30 $$ \limsup_{t\to\infty} \int_{T}^{t} \biggl(\varphi (s)Q(s)- \frac{ (\varphi_{+}'(s) )^{\alpha+1}r(\sigma(s))}{ (\alpha+1)^{\alpha+1}f_{n}(\rho)\varphi^{\alpha}(s) (\sigma '(s) )^{\alpha}} \biggr)\,\mathrm {d}s= \infty, $$

*where* $f_{n}(\rho)$ *is defined by* (26) *and* $\varphi'_{+}(t) = \max\{ 0, \varphi'(t) \}$, *then* (*E*) *is oscillatory*.

### Proof

Assume that (*E*) has a nonoscillatory solution $x(t)$ on $[t_{0},\infty)$. Without loss of generality, we may assume that there exists a $t_{1}\ge t_{0}$ such that $x(t)>0$, $x(\tau(t))>0$, and $x(\sigma (t))>0$ for $t\ge t_{1}$. Define the Riccati function by

31 $$ w(t): = \varphi(t)r(t) \biggl(\frac{z'(t)}{z(\sigma(t))} \biggr)^{\alpha}, \quad t\ge t_{1}. $$

Then $w(t)>0$ for $t\ge t_{1}$. Differentiating (31), we arrive at

32 $$ w'(t) = \frac{\varphi'(t)}{\varphi(t)}w(t) +\varphi(t) \frac{ (r(t) (z'(t) )^{\alpha})'}{z^{\alpha}(\sigma(t))}- \alpha\varphi(t)\sigma'(t)r(t) \biggl( \frac{z'(t)}{z(\sigma (t))} \biggr)^{\alpha}\frac{z'(\sigma(t))}{z(\sigma(t))}. $$

It follows from Lemma 4 that there exists a $T\ge t_{1}$ large enough such that

33 $$ \frac{z'(\sigma(t))}{z'(t)}\ge \biggl(\frac{f_{n}(\rho )r(t)}{r(\sigma(t))} \biggr)^{1/\alpha}, \quad t\ge T. $$

By virtue of (11) and (33), applications of (31) and (32) yield

34 $$ w'(t)\le -\varphi(t)Q(t)+ \frac{\varphi_{+}'(t)}{\varphi(t)}w(t)- \frac{\alpha f_{n}^{1/\alpha}(\rho) \sigma'(t)}{ (\varphi (t)r(\sigma(t)) )^{\frac{1}{\alpha}}}w^{(\alpha+1)/\alpha}(t). $$

Letting

$$ A: = \frac{\varphi'_{+}(t)}{\varphi(t)} \quad \text{and} \quad B := \frac{\alpha f_{n}^{1/\alpha}(\rho)\sigma'(t)}{ (\varphi (t)r(\sigma(t)) )^{\frac{1}{\alpha}}} $$

in (8), it follows now from Lemma 2 and (34) that

35 $$ w'(t)\le - \varphi(t)Q(t)+\frac{ (\varphi'_{+}(t) )^{\alpha+1}r(\sigma(t))}{ (\alpha+1)^{\alpha+1}f_{n}(\rho)\varphi ^{\alpha}(t) (\sigma'(t) )^{\alpha}}. $$

Integrating (35) from *T* to *t*, we obtain

$$ \int_{T}^{t} \biggl(\varphi(s)Q(s)- \frac{ (\varphi'_{+}(s) )^{\alpha+1}r(\sigma(s))}{ (\alpha+1)^{\alpha+1}f_{n}(\rho)\varphi ^{\alpha}(s) (\sigma'(s) )^{\alpha}} \biggr)\,\mathrm {d}s \le w(T), $$

which contradicts condition (30). This completes the proof. □

### Remark 2

Theorem 5 is new because of the constant $f_{n}(\rho)$ (for some $n\ge 0$) appearing in (30). So far, all results obtained in a similar manner have been formulated for $n=0$; see, e.g., [16, 17, 20–23, 26, 27, 36, 37]. Thus, for any given $n>0$, our result essentially improves the previous ones.

Letting $\varphi(t)= R^{\alpha}(\sigma(t)) $ in (30), Theorem 5 yields the following result.

### Corollary 2

*Let condition* (1) *hold and assume that* $\sigma\in\mathrm{C}^{1}([t_{0},\infty),\mathbb {R})$, $\sigma'(t)>0$, *and condition* (27) *holds for some* $\rho>0$. *If*, *for some sufficiently large* $T\ge t_{1}$ *and for some* $n\ge0$,

36 $$ \limsup_{t\to\infty} \int_{T}^{t} \biggl(R^{\alpha}\bigl(\sigma (s) \bigr)Q(s)- \biggl(\frac{\alpha}{\alpha+1} \biggr)^{\alpha+1}\frac {\sigma'(s)}{ f_{n}(\rho)R(\sigma(s))r^{1/\alpha}(\sigma(s)) } \biggr)\,\mathrm {d}s= \infty, $$

*where* $f_{n}(\rho)$ *is defined by* (26), *then* (*E*) *is oscillatory*.

### Example 2

As in Example 1, we consider ($E_{x}$). If we assume that $\rho\le1/\mathrm {e}$ in ($C_{1}$), then the sequence $\{f_{n}(\rho)\}_{n = 0}^{\infty}$ defined by (26) has a finite limit (28), which can be expressed as

$$ f(\rho) = \lim_{n\to\infty}f_{n}(\rho) = - \frac{W(-\rho )}{\rho}, $$

where *W* standardly denotes the principal branch of the Lambert function; see [42] for details. Then, by Corollary 2, ($E_{x}$) is oscillatory if 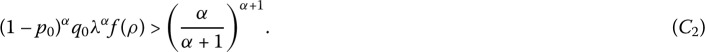 In order to illustrate the efficiency of the above criterion, we stress that an application of (5) yields that condition 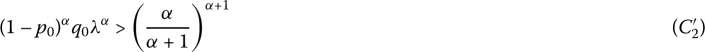 ensures oscillation of ($E_{x}$). For a particular case of ($E_{x}$), equation

37 $$ \bigl( \bigl(x'(t) \bigr)^{3} \bigr)'+\frac{0.3}{t^{4}}x^{3}(0.9t)=0, $$

condition ($C_{2}$) gives $3.5876>0.3164$, which implies that (37) is oscillatory. However, one may see that the left-hand side of inequality ($C_{2}'$) becomes 0.2187, which means that condition ($C_{2}'$) fails to hold for (37). Moreover, one can easily verify that the criterion resulting from Theorem 3 cannot be applied to (37).

The following theorem serves as an alternative to Theorem 5.

### Theorem 6

*Let condition* (1) *be satisfied and assume that there exists a function* $\psi\in\mathrm{C}^{1}([t_{0},\infty),(0,\infty))$ *such that*, *for some sufficiently large* $T\ge t_{1}$,

38 $$ \limsup_{t\to\infty} \int_{T}^{t} \biggl( \psi(s) Q(s)\hat {R}(s) - \frac{ (\psi'_{+}(s) )^{\alpha+1}r(s)}{ (\alpha +1)^{\alpha+1}\psi^{\alpha}(s) } \biggr)\,\mathrm {d}s= \infty, $$

*where* $\psi'_{+}(t) = \max\{ 0, \psi'(t) \}$. *Then* (*E*) *is oscillatory*.

### Proof

Assume that (*E*) has a nonoscillatory solution $x(t)$ on $[t_{0},\infty)$. Without loss of generality, we can suppose that there exists a $t_{1}\ge t_{0}$ such that $x(t)>0$, $x(\tau(t))>0$, and $x(\sigma (t))>0$ for $t\ge t_{1}$. Define the Riccati function by

39 $$ w(t) := \psi(t)r(t) \biggl(\frac{z'(t)}{z(t)} \biggr)^{\alpha}, \quad t\ge t_{1}. $$

Then $w(t)>0$ for $t\ge t_{1}$ and

40 $$ w'(t) = \frac{\psi'(t)}{\psi(t)}w(t) +\psi(t) \frac{ (r(t) (z'(t) )^{\alpha})'}{z^{\alpha}(t)}- \alpha\psi (t)r(t) \biggl(\frac{z'(t)}{z(t)} \biggr)^{\alpha+1}. $$

As in the proof of Theorem 3, we get (16), i.e.,

$$ z(t)\ge\tilde{R}(t)r^{1/\alpha}(t)z'(t) $$

or

$$ \frac{z'(t)}{z(t)}\le\frac{1}{\tilde{R}(t)r^{1/\alpha}(t)}. $$

Integrating the latter inequality from $\sigma(t)$ to *t*, we obtain

41 $$ \frac{z(\sigma(t))}{z(t)}\ge\exp \biggl( - \int_{\sigma (t)}^{t}\frac{\mathrm{d} s}{\tilde{R}(s)r^{1/\alpha}(s)} \biggr). $$

Combining (11) and (41), it follows that

$$\begin{aligned} \frac{ (r(t) (z'(t) )^{\alpha})'}{z^{\alpha}(t)} &\le- Q(t) \biggl(\frac{z(\sigma(t))}{z(t)} \biggr)^{\alpha}\\ & \le- Q(t) \exp \biggl( - \alpha \int_{\sigma(t)}^{t}\frac{\mathrm{d} s}{\tilde{R}(s)r^{1/\alpha}(s)} \biggr) \\ & = - Q(t)\hat{R}(t). \end{aligned}$$

Hence, by (39) and (40), we deduce that

42 $$ w'(t)\le\frac{\psi'_{+}(t)}{\psi(t)}w(t) - \psi(t) Q(t) \hat{R}(t)- \frac{\alpha}{ (\psi(t)r(t) )^{1/\alpha}} w^{(\alpha +1)/\alpha}(t). $$

Letting

$$ A: = \frac{\psi'_{+}(t)}{\psi(t)} \quad \text{and}\quad B := \frac {\alpha }{ (\psi(t)r(t) )^{\frac{1}{\alpha}}} $$

in (8), it follows from Lemma 2 and (42) that

43 $$ w'(t)\le - \psi(t) Q(t) \hat{R}(t)+ \frac{ (\psi'_{+}(t) )^{\alpha+1}r(t)}{ (\alpha+1)^{\alpha+1}\psi^{\alpha}(t) }. $$

Let $T\ge t_{1}$ be sufficiently large. Integrating (43) from *T* to *t*, we have

$$ \int_{T}^{t} \biggl( \psi(s) Q(s) \hat{R}(s)- \frac{ (\psi '_{+}(s) )^{\alpha+1}r(s)}{ (\alpha+1)^{\alpha+1}\psi^{\alpha}(s) } \biggr)\,\mathrm {d}s \le w(T), $$

which contradicts condition (38). The proof is complete. □

### Example 3

As in Example 1, we consider ($E_{x}$). By Theorem 6, ($E_{x}$) is oscillatory if 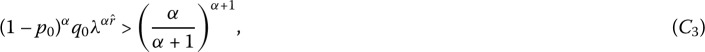 where $\hat{r} := (\alpha/(\alpha+(1-p_{0})^{\alpha}q_{0} \lambda ^{\alpha}) )^{\alpha}$. An application of (6) yields that ($E_{x}$) is oscillatory provided that

$$ (1-p_{0})^{\alpha}q_{0} \lambda^{\alpha}> \biggl(\frac{\alpha}{\alpha +1} \biggr)^{\alpha+1}. $$

It is easy to see that $\hat{r}<1$, and thus our criterion ($C_{3}$) provides a stronger result.

## Conclusions

In the present paper, we have studied the oscillatory behavior of the second-order half-linear neutral delay differential equation (*E*). As it has been illustrated through several examples, the results obtained improve a large number of the existing ones. Our technique lies in establishing some sharper estimates relating a nonoscillatory solution with its derivatives in the case when criteria analogous to ($1_{a}'$)–($1_{b}'$) fail to apply.

The results presented in this paper strongly depend on the properties of first-order delay differential equations. An interesting problem for further research is to establish different iterative techniques for testing oscillations in (*E*) independently on the constant $1/\mathrm {e}$.
